# Persistent repair intermediates induce senescence

**DOI:** 10.1038/s41467-018-06308-9

**Published:** 2018-09-25

**Authors:** F. M. Feringa, J. A. Raaijmakers, M. A. Hadders, C. Vaarting, L. Macurek, L. Heitink, L. Krenning, R. H. Medema

**Affiliations:** 1 Oncode Institute, Division of Cell Biology, Netherlands Cancer Institute, 1066CX Amsterdam, The Netherlands; 2 Oncode Institute, Center for Molecular Medicine, University Medical Center Utrecht, Utrecht University, 3584 CG, Utrecht, The Netherlands; 3 Laboratory of Cancer Cell Biology, Institute of Molecular Genetics, Academy of Sciences of the Czech Republic, 142 20 Prague 4, Prague, Czech Republic; 4 Present Address: ACRF Stem Cells and Cancer Division, Walter and Eliza Hall Institute of Medical Research, Parkville, Victoria, 3052 Australia; 5 Present Address: Hubrecht Institute, The Royal Netherlands Academy of Arts and Sciences (KNAW) and University Medical Center Utrecht, 3584CT Utrecht, The Netherlands

## Abstract

Double-stranded DNA breaks activate a DNA damage checkpoint in G2 phase to trigger a cell cycle arrest, which can be reversed to allow for recovery. However, damaged G2 cells can also permanently exit the cell cycle, going into senescence or apoptosis, raising the question how an individual cell decides whether to recover or withdraw from the cell cycle. Here we find that the decision to withdraw from the cell cycle in G2 is critically dependent on the progression of DNA repair. We show that delayed processing of double strand breaks through HR-mediated repair results in high levels of resected DNA and enhanced ATR-dependent signalling, allowing p21 to rise to levels at which it drives cell cycle exit. These data imply that cells have the capacity to discriminate breaks that can be repaired from breaks that are difficult to repair at a time when repair is still ongoing.

## Abstract (web summary)

Cells with damaged DNA can permanently exit the cell cycle during the G2 phase or recover spontaneously entering mitosis. Here the authors reveal that the decision to exit from the cell cycle in G2 is dependent on the presence of repair intermediates associated with homologous recombination.

## Introduction

Cells need to respond to various types of DNA damage to protect the integrity of their genome. When DNA lesions are encountered, the DNA damage response (DDR) activates a checkpoint signalling cascade that will halt cell cycle progression and activate DNA repair. This arrest is particularly important when DNA double-stranded breaks (DSBs) occur in G2 phase, since cells need to prevent cell division in the presence of broken chromosomes as this can lead to loss or gain of genetic material that could cause cell death, or drive transformation^1–4^.

ATM (ataxia-telangiectasia mutated) and ATR (ATM- and Rad3-related) are the central kinases of the DDR^5,6^. Although ATM and ATR recognize distinct forms of DNA damage, both are needed for proper checkpoint activation when DSBs are encountered^7,8^. Upon recruitment to the DNA lesion, ATM and ATR activate their target kinases Chk2 and Chk1^5,6^, respectively, and promote recruitment of DNA repair proteins to DSB sites^5,6^. The primary repair pathways for DSBs are non-homologous end-joining (NHEJ) and homologous recombination (HR). NHEJ, the more rapid but less accurate of the two, is the most widely used repair mechanism throughout the cell cycle. The relatively easy re-ligation of a DSB by NHEJ does not need extensive processing of the DNA around the DSB. HR on the contrary is restricted to S/G2 phase when a sister homologue is present that can be used as a template for more accurate repair of the DSB. HR-mediated repair requires resection of the DNA at the break site to create extensive single-stranded overhangs that can invade the homologous sister strand. The single-stranded DNA that is created during resection is rapidly covered by the single-strand-binding protein RPA. RPA-coated single-stranded DNA recruits and activates ATR with its co-factor ATRIP^9,10^. RPA needs to be exchanged for Rad51 protein on the single-stranded DNA to start the homology search and complete HR repair^11,12^.

It is still largely unknown how checkpoint (in)activation and repair are coordinated to determine cell fate after DNA damage. We have previously shown that the decision to irreversibly exit the cell cycle is established within a few hours after damage induction in G2 phase, while the ability to recover is retained substantially longer when damage occurs in other phases of the cell cycle^13^. The permanent cell cycle exit from G2 phase is marked by p21-dependent entrapment of Cyclin B1/Cdk in the nucleus, keeping it refractory to re-activation^13,14^. In non-transformed p53-proficient cells this results in induction of senescence^13,15^, and this response is clearly dose-dependent^13,16^, suggesting that the amount of damage is the primary determinant. However, we did observe a clear heterogeneity in cell fate when these same cells were irradiated with a dose of ionizing radiation (IR) they can easily recover from (doses between 0 and 4 Gy of IR). At these lower doses, we can find examples of cells with <10 breaks that permanently withdraw from the cell cycle, versus examples of cells with >20 breaks that recover. This indicates that the number of breaks a cell encounters in G2 phase cannot be the sole determinant for its fate. Therefore, it remains unclear what dictates the decision to enter senescence.

Here we find that the cells that permanently withdraw from the cell cycle display a significant increase in RPA-coated DNA damage foci at 3 h following damage induction, a time when repair is still ongoing. This increase in RPA foci is not paralleled by an increase in Rad51 foci, suggesting that these stretches of resected DNA fail to properly engage in HR-mediated repair. The presence of the structures is associated with enhanced activation of ATR at the moment the decision to exit the cell cycle is made. In line with this, we find that interventions that cause an increase in HR repair intermediates lead to an increase in the percentage of G2 cells that withdraws from the cell cycle via nuclear entrapment of Cyclin B1. These results show that cell fate decisions can be triggered by the detection of damage that will be difficult to repair, already in the first hours after damage induction, at a time when other breaks are still being repaired.

## Results

### Spontaneous recovery awaits successful repair

**Fig. 1:**
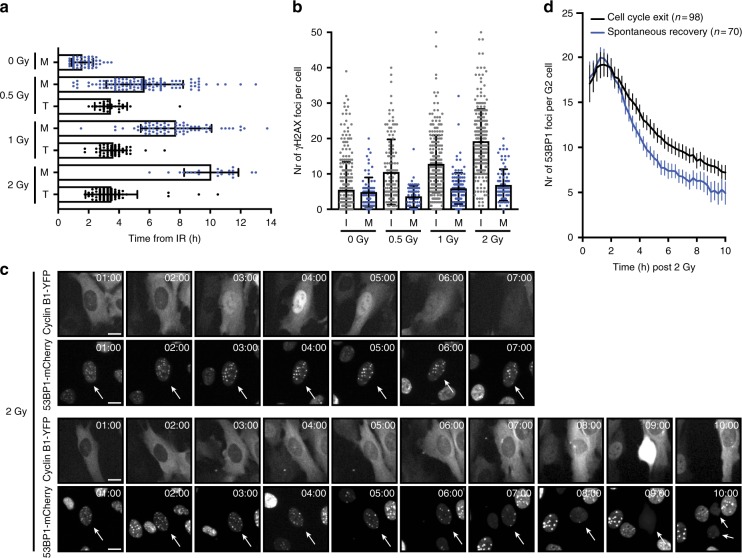
Spontaneous recovery awaits successful repair. **a** Timing of cell cycle exit by onset of Cyclin B1 translocation (T) or spontaneous recovery by mitotic entry (M) after IR with indicated doses. Pooled cells from three independent exp. *n* > 20 Mean + sd. **b** Number of γH2AX foci in interphase (I) cells 1 h after IR and recovering (mitotic, M) cells collected 5–7 h after IR with indicated doses. Pooled cells from three independent exp. *n* > 65 + sd. **c** Stills representing RPE *CCNB1*^YFP^ cells that enter mitosis or that translocate Cyclin B1 after IR (2 Gy) and the corresponding 53BP1^mCherry^ signal in the nucleus. Scale bar 10 μm. **d** Quantification of 53BP1 foci from live cell imaging at indicated timepoints after IR in cells that recover spontaneously and cells that translocate Cyclin B1. *n* = number of cells pooled from three independent experiments + 95% confidence interval

We have previously shown that the decision to permanently withdraw from the cell cycle in G2 phase is marked by nuclear translocation of Cyclin B1, which occurs ~3 h after the damaging insult^13^. This translocation is invariably followed by proteasomal degradation of Cyclin B1 a few hours later and occurs exclusively in the cells that exit the cell cycle and become senescent, while Cyclin B1 remains cytoplasmic in cells that can eventually recover from the arrest^13^. This provides us with an easy assay to monitor cell fate decisions in G2 phase. Non-transformed RPE-1 cells can recover from irradiation with doses up to 4 Gy, while at 5 Gy all cells exit the cell cycle^13^. Using RPE-1 cells expressing endogenously YFP-tagged Cyclin B1 (RPE-*CCNB1*^YFP^) we monitored the timing of spontaneous recovery and cell cycle exit after different low doses of IR. Only cells damaged in G2 phase were included based on Cyclin B1-YFP intensity at the time of irradiation (Supplementary Fig. 1a). The duration of the G2 arrest in cells that do eventually recover increases with increasing doses of IR (Fig. 1a, blue dots). Indeed, and not surprisingly, increasing levels of initial damage are detected in G2 cells upon increasing irradiation dose (Fig. 1b, grey dots). These levels have returned to background levels in recovering cells that enter mitosis, regardless of the initial dose of damage (Fig. 1b, blue dots). This indicates that recovery is limited to cells that have successfully repaired the DNA damage that was inflicted. However, in the cells that do not recover from the arrest, the time of Cyclin B1 translocation is independent of the IR dose, always occurring around 3–4 h after the damaging insult (Fig. 1a, black dots), implying that cells respond to defects in repair well before repair has been completed (Supplementary Fig. 1b). Thus, while the levels of damage influence the duration of the G2 arrest, they do not affect the timing of the decision to permanently exit the cell cycle.

### The decision to exit the cell cycle is not determined by the number of breaks induced

To confirm that the decision to permanently withdraw from the cell cycle is taken before repair is completed, whereas the duration of the arrest corresponds to the time required for repair, we followed RPE-*CCNB1*^YFP^ cells expressing 53BP1-mCherry by time-lapse analysis. Using these cells, we can monitor the progress of DNA repair by analysing the appearance and disappearance of 53BP1 foci, and we can monitor cell fate decisions by analysing the nuclear translocation of Cyclin B1. This analysis confirms that repair is not yet completed at the time when we detect Cyclin B1 translocation (compare Fig. 1c and d). Interestingly, when separating the recovering from the non-recovering cells we could not observe a difference in the maximum number of 53BP1 foci formed after irradiation with 2 Gy (Fig. 1c, d). Differences in the relative numbers of 53BP1 foci do start to appear between 3 and 4 h after the damage (Fig. 1d), but complete resolution of the foci takes much longer, also in the cells that recover. 53BP1 foci numbers detected in live cell imaging correspond to γH2AX foci numbers quantified in fixed cells (Supplementary Fig. 1c). Live cell imaging confirmed that the difference in 53BP1 foci number does not arise through new foci formation, since the average residence time of individual foci is longer in cells that exit G2 after translocation of Cyclin B1 compared to cells that recover spontaneously following irradiation with 2 Gy (Supplementary Fig. 1d). In fact, the damage-associated foci remain visible well after Cyclin B1 has translocated. Together these results imply that the fate of a G2 cell that is irradiated with an intermediate dose of 2 Gy cannot simply be predicted by the number of DSBs that are induced. Rather, its fate seems to be determined by the presence of breaks that will be difficult to repair, at a time when breaks that can more easily be repaired are also still present. This raises the intriguing question how a cell can detect such difficult-to-repair breaks for a decision-making process that takes place at a time that reparable breaks are still abundantly present.

### ATR signalling is the main driver of cell cycle exit in G2 phase

**Fig. 2:**
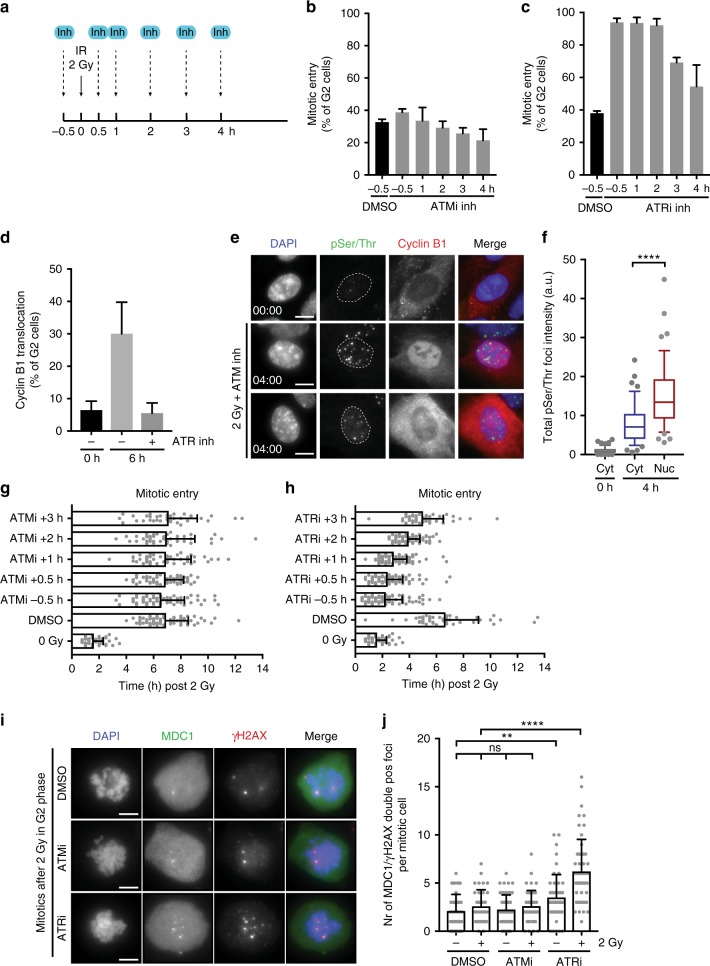
ATR signalling is the main driver of cell cycle exit in G2 phase. **a** Schematic representation of the experimental set-up. **b**, **c** Mitotic entry was scored in RPE *CCNB1*^YFP^-positive (G2)-cells after IR (2 Gy). ATM inhibitor (KU55933) (**b**) or ATR inhibitor (VE-821) (**c**) was added at indicated timepoints before or after IR. Average of three independent experiments + sem. **d** Percentage of G2 cells with nuclear translocated Cyclin B1 at 6 h after OHT addition. S phase cells were excluded based on Edu staining. Prophase cells with nuclear Cyclin B1 localization were excluded based on DNA condensation as stained by DAPI. Average of three independent experiments + sem. **e** Stills represent G2 cells (Edu negative) 0 h or 4 h after IR (2 Gy) with nuclear translocated or cytoplasmic Cyclin B1 localization. pSer/Thr staining in foci represents ATR substrate phosphorylation. Scale bar 10 μm. **f** Quantification of total pSer/Thr phosphorylation signal in foci at indicated timepoint after IR in cells with cytoplasmic or nuclear Cyclin B1 localization. Pooled cells from three independent experiments. *n* > 44. Box plot whiskers indicate 10–90% boundary. ****p < 0.0005 two-sided *t*-test. **g**, **h** Time of mitotic entry after IR (2 Gy) in G2 cells scored in (Fig. 2b, c). Pooled cells from three independent experiments *n* > 35 + sd. **i** Stills represent mitotic cells collected by nocodazole and stained for MDC1 and γH2AX to quantify DSBs in cells that entered mitosis within 9 h after IR (2 Gy) in G2 phase. Cells in S phase at the time of IR are excluded by EdU staining. Scale bar 5 μm. **j** Number of MDC1 and γH2AX double positive foci in mitotic cells as shown in **i**. Pooled cells from two independent experiments *n* > 35 + sd

It is well known that both ATM and ATR are activated when DSBs are introduced in a cell in G2 phase, however a clear understanding of their individual roles in cell fate decisions is lacking. As expected, addition of an ATM inhibitor (KU55933) efficiently blocked IR-induced phosphorylation of Chk2 and Kap1 (Supplementary Fig. 2a), while addition of an ATR inhibitor (VE-821) inhibited IR-induced phosphorylation of Chk1 (Supplementary Fig. 2a). Addition of the ATM inhibitor at 30 min before IR led to a minor increase in cells entering mitosis and a reduction in cells translocating Cyclin B1 (Fig. 2a, b and Supplementary Fig. 2b). This decrease is not unexpected in light of the well-established role for ATM in the initiation of checkpoint signalling and the activation of p53. However, addition of the ATM inhibitor at 1 h after IR could no longer promote mitotic entry, nor could it prevent the translocation of Cyclin B1 (Fig. 2a, b and Supplementary Fig. 2b). In contrast, ATR inhibition before or up to 2 h after IR could fully prevent nuclear translocation of Cyclin B1 and overcome the decision to withdraw from the cell cycle as evidenced by nearly 100 percent mitotic entry of the damaged G2 cells (Fig. 2a, c and Supplementary Fig. 2b). Therefore, ATR activity appears to be most crucial for cell fate decisions in G2 under these conditions, while ATM activity contributes to the initiation of the G2 checkpoint but has little influence on cell fate decisions in G2 phase. The effect of ATR is completely dependent on its effector kinase Chk1, since inhibition of Chk1 (UCN-01 and CHIR-124) likewise prevented nuclear translocation of Cyclin B1 and promoted mitotic entry in damaged G2 cells (Supplementary Fig. 2c). To show that these effects are not limited to RPE-1 cells, we made use of BJ-hTert Fucci cells, that display a similar mode of IR-induced senescence^13,16^. Indeed, also in these non-transformed cells, cell cycle withdrawal in G2 is dependent on ATR (Supplementary Fig. 2d).

It has previously been shown that activation of ATR signalling can drive cells into senescence^17^. Given the crucial role we observe for ATR in determination of cell fate in G2 phase, we tested whether mere ATR activation in the absence of DNA damage could be sufficient to drive nuclear translocation of Cyclin B1. To activate ATR in G2 cells, we created RPE-1 cells expressing a TopBP1^ER^ construct that specifically activates ATR in the nucleus upon 4-hydroxytamoxifen (OHT) addition^17^. As expected, treatment of the RPE-TopBP1^ER^ cells with OHT led to an increase in pan-nuclear γH2AX phosphorylation which plateaued 4 h after OHT addition (Supplementary Fig. 2e). G2/M progression of asynchronously growing cells started to drop after 4 h of OHT treatment, while this was unaffected when OHT treatment was combined with ATR inhibition (Supplementary Fig. 2f). Quantification of Cyclin B1 localization in G2 cells 6 h after OHT addition, shows that ~30% of the cells translocated Cyclin B1 to the nucleus, and this was completely suppressed by the addition of an ATR inhibitor (Fig. 2d). Thus, ATR activation can indeed drive cell cycle exit in G2 cells via nuclear translocation of Cyclin B1.

These data imply that cell to cell variation in ATR signalling in the first 4 h after DNA damage should correlate to specific cellular fate of the individual G2 cells. To confirm this we used a phospho-specific antibody recognizing a large variety of ATM/ATR substrates (pSer/Thr) in the nucleus. To selectively detect ATR-dependent phosphorylation, we included an ATM inhibitor in these experiments. Cells that were in S phase at the moment of irradiation were excluded from the analysis based on Edu staining. We find a striking increase in the level of ATR-dependent substrate phosphorylation in G2 cells with nuclear Cyclin B1 at 4 h after irradiation, as compared to the cells with cytoplasmic Cyclin B1 (Fig. 2e, f). Together these results show that enhanced ATR signalling at early time-points after irradiation, while repair is still ongoing, can drive cell fate decisions in G2 phase.

Consistent with an early ATR-dependent cell cycle exit, we find that addition of an ATR inhibitor rapidly drives G2 cells into mitosis, immediately reverting the damage-induced arrest (Fig. 2h). In contrast, addition of an ATM inhibitor either before or after IR did not affect the duration of the DNA damage-induced G2 arrest (Fig. 2g), indicating that ATR, rather than ATM monitors the progress in DNA repair during G2. Both ATM and ATR inhibition did not alter the speed of G2/M progression in non-irradiated cells (Supplementary Fig. 2g). We did observe a short delay in mitotic entry of irradiated cells treated with the ATR inhibitor compared to non-irradiated cells (Fig. 2h), suggesting that ATM might play a minor role in timing the arrest. Indeed, combining both inhibitors further reduced the delay in mitotic entry of irradiated G2 cells (Supplementary Fig. 2h). Thus, although ATM is known to stimulate resection and ATR signalling upon DSB induction, ATM-independent activation of ATR must be possible, as also observed by others^18–22^. ATR activation and nuclear translocation of Cyclin B1 are somewhat reduced when cells were treated with ATM inhibitor, but the remaining ATR activity can prevent progression to mitosis (Supplementary Fig. 2a, b and Fig. 2g). This suggests that low levels of ATR activity are sufficient to prevent mitotic entry until repair is complete, while higher levels promote a permanent cell cycle exit.

The direct reversal of the G2 arrest upon ATR inhibition suggests that these cells will enter mitosis with unrepaired DSBs, as time for repair was lacking. Therefore, we analysed the number of γH2AX and MDC1 double-positive foci in cells that entered mitosis after irradiation in G2 phase. EdU was added just prior to irradiation to allow us to exclude S phase cells (Supplementary Fig. 2i). Indeed, while ATM-inhibited cells had reduced the number of DSB-foci back to background levels before entering mitosis, ATR-inhibited cells entered mitosis with a significant number of unrepaired DNA breaks (Fig. 2i, j). A similar increase was detected in the number of 53BP1-mCherry foci in cells 1 h prior to mitotic entry (Supplementary Fig. 2j). Interestingly, a slight increase in the number of breaks in mitotic cells was already observed in non-irradiated cells treated with the ATR inhibitor, again underlining the importance of ATR signalling to monitor repair and prevent mitotic entry in damaged G2 cells (Fig. 2j). Thus, we propose that sustained ATR activity during the first 4 h after damage induction drives cells out of the cell cycle via nuclear translocation and degradation of Cyclin B1, resulting in the induction of senescence in cells with a 4N DNA content. In addition, our data show that sustained ATR activity is necessary to prevent mitotic entry of G2 cells with unrepaired breaks. We find that a majority of the ATR inhibited G2 cells that went through mitosis in the presence of DNA damage do not enter a new cell cycle within 48 h and show reduced colony outgrowth (Supplementary Fig. 2k, l). This indicates that while ATR signalling is required for induction of senescence in G2 phase, ATR-independent mechanisms do exist to drive cells out of the cycle in the subsequent G1. But important to note that despite this back-up, a proportion of the damaged G1 cells that progressed through mitosis after ATR inhibition manage to grow out (Supplementary Fig. 2k,l). This means that the failure to promote a cell cycle exit in G2 when difficult-to-repair breaks are detected, results in an enhanced risk to escape senescence and propagate cells with a compromised genetic integrity.

### Timely negative feedback is crucial to prevent irreversible cell cycle exit in G2 phase

**Fig. 3:**
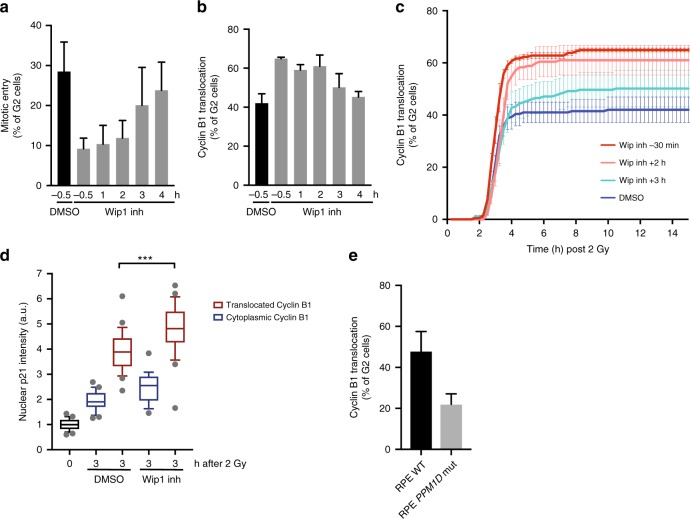
Timely negative feedback is crucial to prevent irreversible cell cycle exit in G2 phase. **a** Mitotic entry scored in RPE *CCNB1*^YFP^ cells within 15 h following IR (2 Gy). Wip1 inh or DMSO were added at indicated timepoints before or after IR. Average of three independent experiments + sem. **b** Percentage of cells that translocate Cyclin B1 following IR treated as in **a**. Average of three independent experiments + sem. **c** Cumulative onset of nuclear translocation of Cyclin in cells treated in **b**. Average of three independent experiments + sem. **d** Nuclear p21 intensity in G2 cells 3 h after 2 Gy in presence or absence of Wip1 inhibition. Cells that translocated Cyclin B1 to the nucleus were separated from cells with cytoplasmic nuclear B1 localization. *n* > 18 cells from representative experiment. Box plot whiskers indicate 10–90% boundary. ***p < 0.005 two-sided *t*-test. **e** Percentage of G2 cells that translocate Cyclin B1 to the nucleus and exit the cell cycle following IR (2 Gy) in RPE *CCNB1*^YFP^ cells with wild-type or truncated Wip1. Average of three independent experiments + sem

Checkpoint signalling is intrinsically limited through the action of several negative feedback loops that act to dampen activation of checkpoint kinases and the induction of p53. One essential negative regulator of the DNA damage checkpoint in G2 phase is the *PPM1D*/Wip1 phosphatase^23^, responsible for dephosphorylation of ATM and ATR substrates, including p53 and ATM and ATR themselves^24–26^. Wip1 is a direct transcriptional target of p53, causing activation of this negative feedback to be enforced by the induction of transcription of Wip1. Addition of a selective small molecule inhibitor of Wip1 (GSK2830371) right before irradiation resulted in a clear increase in the fraction of cells that translocated Cyclin B1 to the nucleus, combined with a concomitant increase in the fraction of cells that exits from the cell cycle (Fig. 3a–c). Thus, negative regulation of checkpoint signalling by Wip1 is already required during the first hours of the damage response to prevent a permanent cell cycle exit, while the timing of cell cycle exit is not affected by the loss of Wip1 (Fig. 3c). This suggests that, p53/p21 accumulation needs to be paralleled with Wip1 induction to provide an opportunity for spontaneous recovery. Indeed, we find that Wip1 inhibition results in higher levels of p21 as early as 3 h after irradiation (Fig. 3d). This can explain the increase in G2 cells that exit via translocation of Cyclin B1, since the depletion of p21 completely reverts the reduced recovery observed upon Wip1 inhibition (Supplementary Fig. 3b). Added 4 h after irradiation, the Wip1 inhibitor did not induce any further increase in translocations, while this still partly prevented recovery (Fig. 3a, b). Taken together, these data indicate that negative regulation of checkpoint signalling by Wip1 is a crucial determinant of cell fate, implying that the cells that withdraw from the cell cycle do so because they fail to dampen checkpoint signalling in a timely manner.

Truncating mutations in the *PPM1D* gene that lead to Wip1 gain-of-function have been reported in HCT116 and U2OS cell lines as well as cancer patients^27^. To study the effect of such truncation mutants on the G2 cell fate decision, we generated RPE-*CCNB1*^YFP^ cells with a truncated version of Wip1 (RPE-*CCNB1*^YFP^ *PPM1D*^Trunc^) (Supplementary Fig. 3c). Importantly, expression of this truncated isoform results in a strong reduction in the percentage of cells that translocate Cyclin B1 in response to irradiation (Fig. 3e). The accompanied increase in spontaneous recovery marks the importance of tight Wip1 regulation for accurate cell fate decisions in G2 phase (Supplementary Fig. 3d).

The strict timing of G2 exit via translocation of Cyclin B1 corresponds well with the timing of p21 protein accumulation, which follows the oscillatory behaviour of p53 upon DNA damage induction (Supplementary Fig. 3a). p53 levels peak around 2 h after IR before they drop back to background levels 4 h after irradiation (Supplementary Fig. 3a), which could explain why Wip1 inhibition at 4 h does not drive any further translocation of Cyclin B1. Irradiation with a second low dose at 4 h after the initial DNA damaging event, can drive cells that retained Cyclin B1 in the cytoplasm after the first cycle of irradiation, into cell cycle exit via translocation of Cyclin B1 (Supplementary Fig. 3e), implying that the cells that do not reach the p21 threshold within the first oscillation and hence are prone to recover from a DNA damaging event, do not become refractory to additional DNA damage signalling.

### Efficient processing of DNA lesions is necessary to prevent a cell cycle exit

**Fig. 4:**
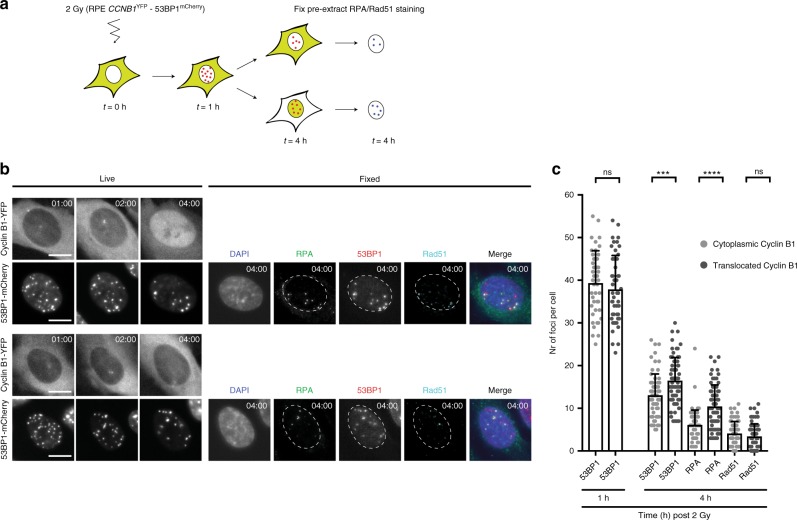
Efficient processing of DNA lesions is necessary to prevent cell cycle exit. **a** Schematic representation of the experimental set-up. **b** Stills represent RPE *CCNB1*^YFP^ cells with 53BP1^mCherry^ tracked by live-cell imaging at indicated timepoints after IR (2 Gy) followed by an image of the identical cell that was fixed 4 h after IR and stained for RPA and Rad51. Scale bar 10 μm. **c** Number of indicated foci in cells that have cytoplasmic Cyclin B1 localization at 4 h after IR (2 Gy) or cells that translocated Cyclin B1 and therefore have nuclear localization at 4 h after IR. 53BP1 foci were determined at indicated timepoints by live-cell imaging of RPE *CCNB1*^YFP^-53BP1^mCherry^ cells. RPA and Rad51 foci were determined in the tracked cell by imaging of the same position after fixation, pre-extraction and antibody staining for RPA and Rad51. Pooled cells from three independent experiments. *n* > 65 + sd

The data presented thus far show that tightly balanced checkpoint signalling in the first hours after irradiation determines the chance to recover and that early detection of difficult-to-repair breaks becomes determinant for the decision to exit or recover in G2 phase. To understand how progress of DNA repair can be detected and translated into an early cell fate decision in the individual G2 cell, we examined the relative use of NHEJ and HR in cells that translocated Cyclin B1 or that retain cytoplasmic Cyclin B1, respectively. We hypothesized that high levels of single-stranded DNA, causing sustained activation of ATR could drive the cell cycle exit. Single-stranded DNA is produced from double-stranded breaks by resection that takes place as part of HR-directed repair, and we reasoned that the relative number of breaks processed by HR repair but also the efficacy of HR repair itself could determine the fate of a damaged G2 cell. To test this hypothesis, we tracked irradiated RPE-*CCNB1*^YFP^ cells expressing 53BP1-mCherry by live cell imaging to distinguish cells that translocate Cyclin B1 from cells that retain cytoplasmic Cyclin B1. At 4 h after damage these cells were fixed and stained for RPA and Rad51, to investigate the extent of HR-mediated repair in each of the fractions (Fig. 4a). Using this experimental set-up, we were able to quantify the number of 53BP1-, RPA- and Rad51 foci in cells that translocated Cyclin B1 within the first 4 h after irradiation versus cells that retained cytoplasmic Cyclin B1 localization during this period (Fig. 4b). Separating the cells according to their Cyclin B1 phenotype we again find no difference in the initial number of 53BP1 foci formed after irradiation, such a difference only becomes apparent at 4 h, confirming our previous results (Fig. 4c). Strikingly, we find a very pronounced difference in the number of RPA-coated DNA damage foci in cells that translocated Cyclin B1 compared to the ones that retained cytoplasmic Cyclin B1. This difference in 53BP1 foci is entirely due to an increase in 53BP1/RPA double-positive foci, indicating that the real difference lies in the number of residual resected DNA breaks (Supplementary Fig. 4a). The difference in RPA-coated DNA breaks was not paralleled by a difference in the number of Rad51 foci (Fig. 4c), suggesting that cells that withdraw from the cycle encounter problems in the exchange of RPA for Rad51, implying that HR-directed repair is initiated in these cells but not efficiently executed.

### Disrupting DNA repair efficiency drives cell cycle exit

**Fig. 5:**
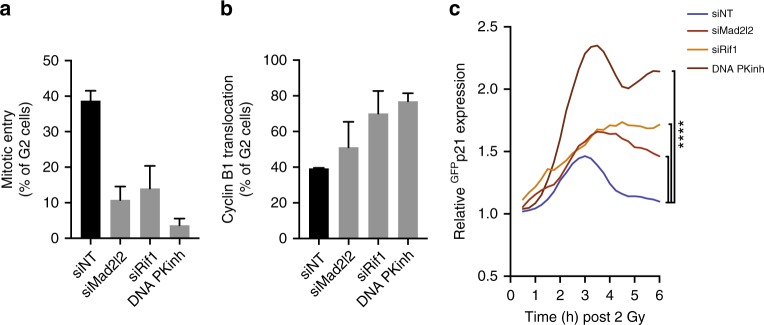
Disrupting DNA repair efficiency drives cell cycle exit. **a** Mitotic entry scored in RPE *CCNB1*^YFP^ cells within 15 h following IR (2 Gy). Average of three independent experiments + sem. **b** Percentage of RPE *CCNB1*^YFP^ cells that translocate Cyclin B1 after IR (2 Gy). Average of three independent experiments + sem. **c** Induction of ^GFP^p21 expression after IR (2 Gy) upon depletion or inhibition of the indicated repair proteins. Mean lines from pooled individual cells from two independent experiments, *n* > 50 cells per condition, significance by 2-way ANOVA

Our data imply that particularly the induction of HR-directed repair results in possible difficulties that can trigger a cell cycle exit. Therefore, we set out to test whether inhibition of NHEJ-directed repair, causing a relative increase in HR-directed repair, could drive cell fate towards cell cycle exit. Indeed, inhibition or knock-down of essential components of NHEJ (DNA-PKcs, Rif1 and Mad2l2) invariably led to a reduction in spontaneous G2 recovery and an increase in the percentage of G2 cells translocating Cyclin B1 (Fig. 5a, b, Supplementary Fig. 4b). In line with this, we observed a delay in the kinetics of 53BP1 foci resolution after IR when NHEJ repair was disturbed (Supplementary Fig. 4c). Moreover, we find that the cells that do recover in presence of the DNA-PK inhibitor are invariably the ones that did repair the damage (Supplementary Fig. 4d).

Since we know that translocation of Cyclin B1 is dependent on p21 induction, we generated RPE-1 cells with endogenously GFP-tagged p21 (RPE-^GFP^*CDKN1A*) to monitor its levels following IR in single cells (Supplementary Fig. 4e,f). Translocation of Cyclin B1 in all our conditions is restricted to a timeframe between 2 and 4 h after IR, which corresponds with the timing of p21 induction detected in our RPE-^GFP^*CDKN1A* cells (Supplementary Fig. 4e). Disruption of NHEJ repair by inhibition of DNA-PK or depletion of Mad2l2 or Rif1 in these cells resulted in a significant increase in p21 induction within 4 h after IR (Fig. 5c), consistent with a higher fraction of cells in which we observe translocation of Cyclin B1 to the nucleus (Fig. 5b). In addition, inhibition of Plk1, known to perturb the exchange of RPA for Rad51^28^, also resulted in enhanced translocation of Cyclin B1 (Supplementary Fig. 4g). Together, these results show a direct link between DNA repair and cell fate in G2 phase to protect genomic stability in non-transformed cells.

## Discussion

The response to DSBs can vary among individual cells within a population, and we have previously shown that a difference in cell cycle stage at the moment of irradiation can affect cellular fate^1,16^. However, heterogeneity in cell fate is also observed in cells that are damaged in the same cell cycle stage. Here we show that the variance in cell fate of G2 cells cannot be explained solely by an absolute difference in the number of DSBs and that cell fate decisions after DNA damage must somehow be affected by qualitative differences that exist between individual breaks. We find that the efficiency of processing DNA breaks is crucial for the fate of a damaged G2 cell. Our data show that not the initial level of checkpoint activation, but the amplitude of ATR-dependent checkpoint signalling that remains at 3–4 h after irradiation determines if recovery is allowed. In addition, we find that ATR activity is necessary throughout the G2 arrest to prevent mitotic entry before DNA repair is completed.

This raises the question how ATR activity is maintained at a high level in the cells that withdraw from the cell cycle, while its activity is quenched, but not completely silenced, in the cells that will recover? Induction of resection drives ATR-dependent checkpoint signalling, but when DSBs are repaired instantly via NHEJ or when resected DNA is rapidly covered by Rad51 to execute HR-dependent repair, signalling through ATR is reversed relatively quickly, leading to limited accumulation of p21 and no translocation of Cyclin B1 to the nucleus. In such a scenario, cells retain Cyclin B1 in the cytoplasm and keep the option to re-activate the G2/M machinery and recover spontaneously at later time-points. The high number of RPA-positive foci that still exist at 4 h after damage-induction in cells that are destined to withdraw from the cell cycle, indicates that residual stretches of ssDNA continue to activate ATR specifically in the non-recovering cells. Interestingly, no difference in the number of Rad51-positive foci was observed in recovering versus non-recovering cells. This suggests that the difficult-to-repair breaks that promote induction of senescence are resected breaks that fail to complete HR-dependent repair because exchange of RPA and Rad51 is impaired. There are several explanations how such problems in repair could arise. First, the problem in repair could arise because the chromatin context of these difficult-to-repair breaks is not favourable for HR-dependent repair. In support of this notion, we find that the remaining RPA-positive foci also stain positive for 53BP1, suggesting continued competition between HR and NHEJ. Alternatively, these RPA-positive foci could correspond to breaks that initially attempted NHEJ-dependent repair, but this failed, after which pathway switching was induced. In such a situation, end resection will be induced with a delay with respect to breaks that immediately engage in HR, causing newly generated stretches of ssDNA to appear resulting in further activation of ATR at later stages of the damage response. This could potentially lead to a problem in recruitment of Rad51, because Plk1 activity, which itself is inhibited upon genotoxic stress, is needed for proper loading of Rad51 during homology-directed repair^28–31^, causing RPA-coated ssDNA to persist. It should be noted however, that Cdk-dependent phosphorylation is needed for end-resection and ATR activation^32–37^, and recent data indicate that residual Cdk1/2 activity is necessary to drive Cyclin B1 translocation^38^.

Whatever the exact scenario to generate these persistent RPA-coated strands, all scenarios would allow a cell to monitor difficulties in repair and allow it to avoid division of cells that contain irreparable damage. This involves persistent ATR activation causing p21 levels to reach a threshold above which nuclear translocation and irreversible inhibition of Cyclin B1/Cdk is inevitable. We would like to stress that this might not be the only route to induction of senescence in a damaged G2 cell. For example, at doses of 5 Gy or higher all RPE-1 cells permanently withdraw from the cell cycle^13^. This might be due to persistent ATR signalling, but one can also imagine that the high number of DSBs causes a level of ATM activation sufficient to induce levels of p21 that can promote nuclear translocation of Cyclin B1. Also, it should be noted that while an override of ATR-induced senescence in G2 does allow cells to enter mitosis, this does not mean that they will continue to proliferate. Only a subset of cells manages to continue to proliferate, while the majority of cells remain blocked in the subsequent G1 phase, indicating that there are alternative ways to withdraw these cells from the cell cycle. Nonetheless, the fraction that does continue to proliferate does so after a mitosis with broken chromosomes, putting it at a high risk of genetic abnormalities. This implies that ATR-dependent cell fate decisions in G2 are relevant to sustain genomic integrity.

The importance of the p53 counteracting phosphatase Wip1 for recovery from DNA damage in G2 phase has been shown before^23^. However, we now find that Wip1 activity is already needed right after initiation of DNA damage to dampen the initial increase in p53 and p21 signalling to influence the cell fate decision process in G2 cells. In accordance, we find that the cell fate decision process is greatly hampered in cells harbouring a stabilizing mutant of Wip1 also found in cancer patients. This further emphasizes the importance of Wip1 for cell fate decisions and shows how mutations in Wip1 can threaten genomic integrity.

It may come as somewhat of a surprise that engagement of HR, which is considered the most accurate mechanism to repair a DSB^39^, increases the chance for a permanent cell cycle exit in G2 phase. However, HR-directed repair involves extensive processing of the DNA, which provides space for formation of complicated intermediate structures. When HR cannot be completed successfully these might drive chromosomal instability in subsequent cell cycles^40^. Therefore, cell cycle exit upon prolonged ATR signalling might simply be a consequence of the detection of difficult-to-repair breaks, rather than a consequence of the detection of HR-related repair intermediates. The prominent activation of ATR that normally occurs in S phase will not trigger senescence because of the constant PCNA-dependent degradation of p21 in S phase, allowing completion of DNA replication and HR-directed repair in S-phase, even when DSBs are present^41–43^. However, when unrepaired breaks continue to signal through ATR in the subsequent G2 phase, they can trigger cell cycle exit via translocation of Cyclin B1.

Taken together, our results show how cells with difficult-to-repair DNA lesions are rapidly eliminated from the cycling population, protecting genomic stability within a population of cells. It will be very interesting to resolve what causes some of the breaks to persist, and what the contributions of the genetic and epigenetic landscape are to this phenomenon.

## Methods

### Cell culture

hTert-immortalized retinal pigment epithelium (RPE-1) cells (ATCC) were maintained in Dulbecco’s Modified Eagle Medium/Nutrient Mixture F-12 (DMEM/F12, Gibco) supplemented with ultraglutamine, penicillin/streptomycin and 10% foetal calf serum. For generation of RPE-1 cells in which a fluorescent tag was introduced in one allele of Cyclin B1 (RPE-*CCNB1*^YFP^) AAV Virus expressing a targeting sequence with 959 bp homology upstream-eYFP- and 1256 bp homology downstream in the *CCNB* gene was collected 2 days after transfection of HEK293 cells with pAAV-eYFP together with pRC and pHelper plasmids. RPE-1 cells were infected with the targeting AAV virus and eYFP positive cells were FACS sorted and plated to collect single cell clones. Clones were selected based on correct eYFP expression on the centrosomes and degradation at the end of mitosis. RPE-*CCNB1*^YFP^-53BP1 mCherry cells were generated using a retroviral 53BP1-mCherry construct. Amphotropic Phoenix cells were transfected with 53BP1-mCherry using X-tremeGENE (Roche) according to manufacturer’s protocol. Virus was harvested after 48 h and used to infect RPE *CCNB1*^YFP^ cells for 24 h. mCherry positive cells were sorted out by FACS 2 weeks later.

RPE-TopBP1^ER^ cells were generated using the retroviral pMX-PIE - TopBP1^ER^-GFP expressing construct (kind gift of dr. Luis Toledo)^17^. Amphotropic Phoenix cells were transfected with the indicated construct using X-tremeGENE (Roche) according to manufacturer’s protocol. Medium was refreshed after 24 h and virus was harvested 48 h later to infect RPE-1 (ATCC) cells using standard procedures. Cells were selected using 2 mg/ml puromycin for 3 days before sorted in a MoFlo Astrios (Beckman Coulter) for GFP expression. Polyclonal GFP expressing cell line was used for experiments described in this manuscript.

RPE-*CCNB1*^YFP^ *PPM1D*^Trunc^ cells were generated using plasmid coding TALENs (obtained from Zgenbio Biotech) targeting tgaagaaaattgcgcta and gatacatgattctttga sequences in exon 6 of the human *PPM1D* gene. Single clones were expanded and were tested for expression of truncated Wip1 by immunoblotting. Targeting of *PPM1D* in exon 6 was confirmed by sequencing of genomic DNA.

RPE-^GFP^p21 cells were generated using a baculoviral delivery strategy (described in ref. ^44^). We used a baculovirus transfer vector encoding 3xFLAG tagged SpCas9 fused to GFP with a 2 A self-cleaving sequence (Cas9-GFP) (Supplementary Fig 5) and introduced a sgRNA targeting the transcriptional start site of *CDKN1A* (exon 1:5′- ATCCCCAGCCGGTTCTGACA-3′) after a U6 promotor in the Cas9-GFP vector. In addition, a homology directed repair (HDR) template was designed flanking the GFP coding sequence with two homology arms (HA), corresponding to chr6:36684104-36685454 (3′RHA) and chr6:36682523-36684104 (5′LHA) (GRCh38) and cloned into pUNKI plasmid. The HDR template was introduced into the baculoviral Cas9-GFP vector using the Not1 sites for subcloning. The Bac-to-Bac system in combination with EMBacY cells was used to generate Bacmids^44^. Baculovirus was produced by transfection of bacmids into Sf9 insect cells^44^. RPE-1 cells were plated in normal culture medium supplemented with baculovirus at an MOI of 25–75 for baculoviral transduction. ATMi (10 µM) was added to suppress the stress response following baculoviral transduction. Medium was exchanged 16 h after infection for normal growth medium and cells were left to recover for 24 h before they were plated single cell per well in a 96-wells plate. Clones were tested for ^GFP^p21 expression by live cell imaging following IR or nutlin treatment.

All cell-lines used were tested negative for mycoplasma contamination.

### Time lapse microscopy and ionizing radiation

Cells were grown in DMEM/F12, supplemented with ultraglutamine, penicillin/streptomycin and 10% foetal calf serum on Lab-Tek II chambered coverglass (Thermo Scientific) for live-cell imaging. Images were obtained using a DeltaVision Elite (applied precision) maintained at 37 °C and 5% CO2 equipped with a 10 0.4 numerical aperture (NA) or 20 0.75 NA or 40 1.35 NA lens (Olympus) and cooled CoolSnap CCD camera. A Gammacell Exactor (Best Theratronics) with a ^137^Cs-source was used for γ-irradiation. Image analysis was performed by custom-written ImageJ software^16^.

### Immunofluorescence

Cells were fixed on coverslips for 15 min at room-temperature using 3.7% paraformaldehyde. Then cells were permeabilized for 5 min using 0.2% Triton X-100 in 1x PBS supplemented with 0.05% Tween-20 (PBS-T). Cells were blocked in PBS-T with 3% bovine serum albumin (BSA) for 1 hour. Antibody incubations were performed overnight at 4 °C or for 2 h at room-temperature. After incubation with secondary antibody coverslips were washed once with PBS-T followed by a wash with EdU staining buffer (100 mM Tris-HCl pH8,5, 1 mM CuSO_4_). EdU was stained by incubation in EdU staining buffer, with 100 mM ascorbic acid and AF-647 azide (Invitrogen, 1/1000) for 30 min at room-temperature. After washing 3 times with PBS-T coverslips were mounted on microscopic slides using Prolong Gold (Invitrogen) and stored at 4 °C. Images were obtained using a DeltaVision Elite (applied precision) equipped with a 10 0.4 numerical aperture (NA) or 20 0.75 NA or 40 1.35 NA lens (Olympus) and cooled CoolSnap CCD camera. Cells that were tracked by live-cell imaging before immunofluorescent staining were pre-extracted by addition of 0.2% Triton X-100 in 1x PBS supplemented with 0.05% Tween-20 (PBS-T) after removal of the medium. Pre-extraction was terminated after 30 s by paraformaldehyde addition at 3.7% final concentration for 15 min. Subsequent antibody staining was performed as described above. Image analysis was performed by custom-written ImageJ software^16^, or manually in case of mitotic cells.

### Antibodies and chemicals

The following primary antibodies were used in this study: anti-γH2AX (ser139p; 05–636 Upstate, 1/500), anti-γH2AX (ser139p; 2577 Cell Signaling, 1/500), anti-Cyclin B1 (GNS1; sc-245 Santa Cruz, 1/500), anti-ATM/ATR phosphor (ser/thr) 4F7 substrate (2909; Cell Signaling, 1/500), anti-MDC1 (ab11171 Abcam, 1/500), anti-p21 (C-19; sc-397, Santa Cruz, 1/500), anti-RPA (ab-2; NA18, Calbiochem, 1/200), anti-Rad51 (H-92; sc-8349, Santa Cruz, 1/500), anti-Cdk4 (C-22; sc-260 Santa Cruz, 1/1,000), anti-pKap1 s824 (A300-7671 Bethyl, 1/1000), anti-pChk1 ser317 (2344 Cell Signaling, 1/500), anti-pChk2 thr68 (2661 Cell Signaling, 1/500), anti-p53 (DO-1; sc-126 Santa Cruz, 1/1000), anti-Wip1 (H300; sc-20712, Tebu, 1/500), anti-GFP (sc-8334, Santa Cruz, 1/1000), anti-HSP90 (sc7947 Santa Cruz, 1/1,000), anti-MAD2B (sc-135977, Santa Cruz, 1/500), anti-Rif1 (A300-569A Bethyl, 1/1000). The following secondary antibodies were used for western blot experiments: peroxidase-conjugated goat anti-rabbit (P448 DAKO, 1/1000) and goat anti-mouse (P0447 DAKO, 1/1000). Secondary antibodies used for immunofluorescence were goat anti-rabbit/Alexa 488 (A_11008 Molecular probes, 1/1000), goat anti-mouse/Alexa 568 (A11004 Molecular probes, 1/1000) and goat anti-rat/Alexa 647 (A21247 Molecular probes, 1/600).

Chemicals used in this study: Nocodazole (Sigma, 250 ng/ml final), ATM inhibitor (KU55933 Merck Millipore, 10 μM final), ATR inhibitor (VE-821 Selleckchem, 5 μM final), Chk1 inhibitor (UCN01 Sigma, 0.3 μM final and CHIR-124 Santa Cruz, 0.5 μM final), DNA PK inhibitor (NU-7441; 14881, Cayman, 1 μM final), Plk1 inhibitor (BI-2536; 1129, Axon, 100 nM final), 4-hydroxytamoxifen (HY-16950; MedChem express/bio-connect, 500 nM final) and Wip1 inhibitor (GSK 2830371, MedChem Express, 10 μM final).

### siRNA information

ON-TARGETplus SMARTpool or set of 4 siRNAs targeting Non-target, CDKN1A, p53, Mad2l2 and Rif1 were from Dharmacon and were transfected using RNAiMAX (Life Technologies) according to manufacturer’s protocol. All transfections were performed 24 h before experiment.

### Sample sizes

For all experiments where phenotypic outcome was quantified at least 50 cells per condition in each independent biological replicate were scored. *n* > 50.

## Electronic supplementary material

Supplementary Information

Peer Review File

## Data Availability

All relevant data are available from the authors upon reasonable request
