# Supplementary material for: Persistent repair intermediates induce senescence
Source: Nat Commun. 2018 Sep 25;9:3923. doi: 10.1038/s41467-018-06308-9 (PMC6156224; doi:10.1038/s41467-018-06308-9)
Supplement: Supplementary file 1 — Supplementary Information [file 41467_2018_6308_MOESM1_ESM.pdf]

Supplementary Figure 1

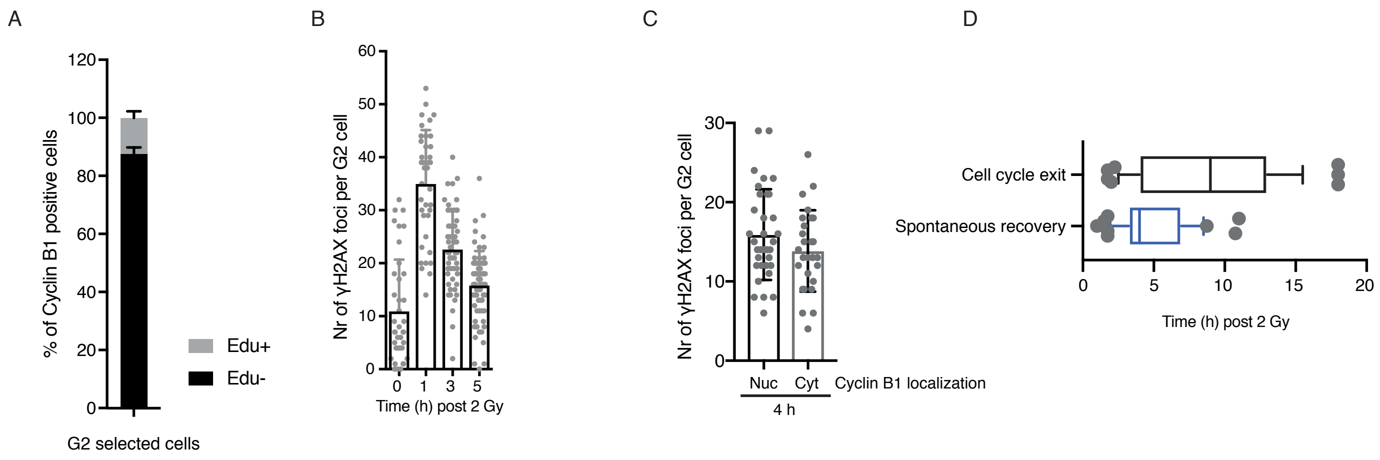

Supplementary figure 1. a) G2 cells in all live-cell experiments were selected based on Cyclin B1-YFP expression. This validation confirms 90% of the Cyclin B1 expressing cells that would be selected as G2 are Edu negative and therefore indeed finished S phase and are true G2 cells. Average of three independent experiments mean + sem **b)** Quantification of yH2AX foci number over time following IR (2 Gy). n>40 cells combined from three independent experiments mean +sd. **c)** Quantification of yH2AX foci number at 4 h after 2 Gy in cells with nuclear vs cytoplasmic Cyclin B1 staining. Pooled cells from 2 independent experiments. n>30 mean +sd. **d)** Quantification of resolution time of individual foci in G2 cells that exit the cell cycle via translocation of Cyclin B1 or that recover spontaneously after IR (2 Gy). n>50 foci from 6 independent cells per condition. Box plot whiskers indicate 10-90% boundary.

Supplementary Figure 2

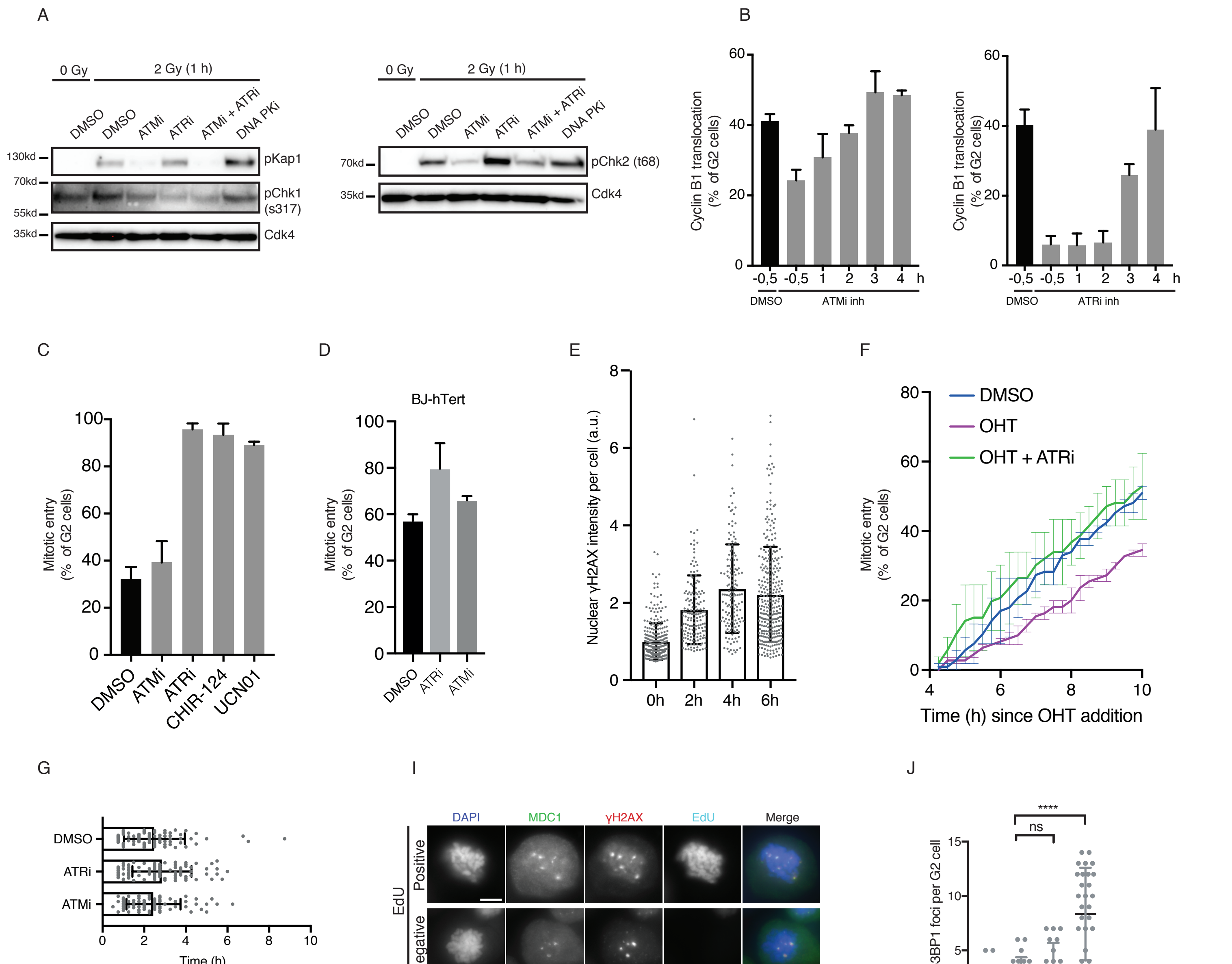

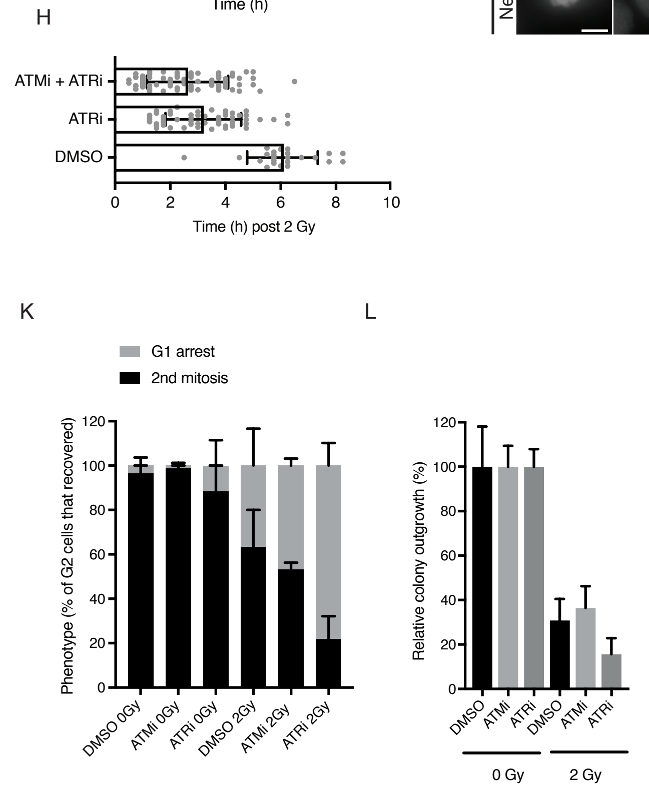

Supplementary figure 2. a) WB showing functional inhibition of ATM and ATR targets by the specific inhibitors. **b)** Percentage of G2 cells that translocate Cyclin B1 and exit the cycle after IR (2 Gy) with indicated inhibition as in (Fig. 2b,c). Average of three independent experiments +sem. **c)** Mitotic entry scored in RPE *CCNB1*^{YFP} cells within 15 h after IR (2 Gy) in presence of indicated inhibitors. Average of three independent experiments +sem **d)** Mitotic entry scored within 20 h after IR (4 Gy) in BJ-hTert cells in presence of indicated inhibitors. Average of two independent experiments + sem. **e)** Quantification of nuclear γH2AX intensity at indicated timepoints after OHT addition in RPE-TopBP1^{ER} cells. Pooled cells from two independent experiments. n>150 cells per condition. mean + sd. **f)** Cumulative mitotic entry of asynchronously growing RPE-TopBP1^{ER} cells starting at 4 h after OHT addition. Average of two independent experiments + sem. **g)** Timing of mitotic entry of undamaged G2 cells after indicated treatment. Pooled cells from 2 independent experiments. n> 70 Mean + sd. **h)** Timing of mitotic entry of G2 cells following IR (2 Gy) in presence of indicated inhibitors. Example of 1 biological replicate n>24 mean +sd. **i)** Stills represent mitotic cells collected by nocodazole that were in S (EdU positive) or in G2 (EdU negative) phase at the time of IR. Only cells damaged in G2 phase (Edu negative) were used for quantification of MDC1 and yH2AX double positive foci in mitosis 9 h after IR (2 Gy) as shown in (Fig. 2f and g). Scale bar 5 µm. **j)** Quantification of 53BP1 foci per nucleus right before mitotic entry after IR (2 Gy) and treatment with indicated inhibitors. Pooled cells from two independent experiments. n>20. Mean + sd. ****p<0,0005 two-sided t-test. **k)** Quantification of G1 daughter cell phenotype of all G2 cells that entered mitosis after indicated treatments. Cells were tracked for 48 h after onset of the experiment. Average of two independent experiments + sem **l)** Clonogenic outgrowth of cells after indicated treatment in G2 phase (thymidine synchronised). Inhibitors were washed out 12 h after IR. Average of two experiments in triplicate + sd.

Supplementary Figure 3

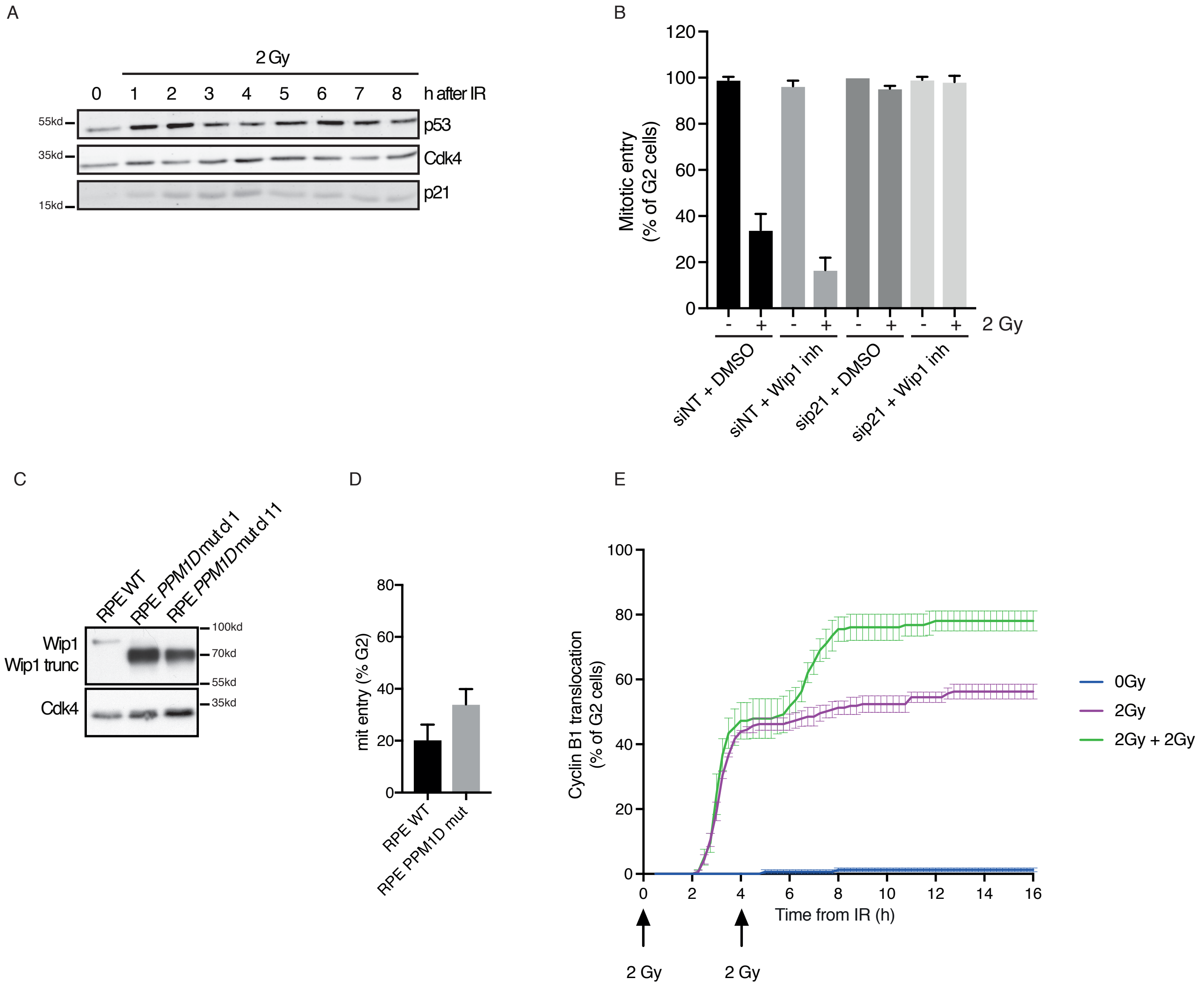

Supplementary figure 3. a) Western blot showing p53 and p21 protein levels at indicated timepoints after IR (2 Gy) in G2 synchronized cells. Representative of three independent experiments. **b)** Spontaneous recovery of G2 cells treated as indicated. Average of two independents + sd **c)** Representative western blot showing overexpression of mutated/truncated Wip1. Clone 11 was used in figure 3e. **d)** Percentage of G2 cells that enter mitosis following IR (2 Gy) in RPE *CCNB1*^{YFP} cells with wild-type or truncated Wip1. Average of three independent experiments + sem. **e)** Cumulative onset of Cyclin B1 translocation to the nucleus in G2 cells following irradiation with 2 Gy. Where indicated cells were treated with a second dose of 2 Gy 4 h after the initial damaging event. Average of three independent experiments + sem.

Supplementary Figure 4

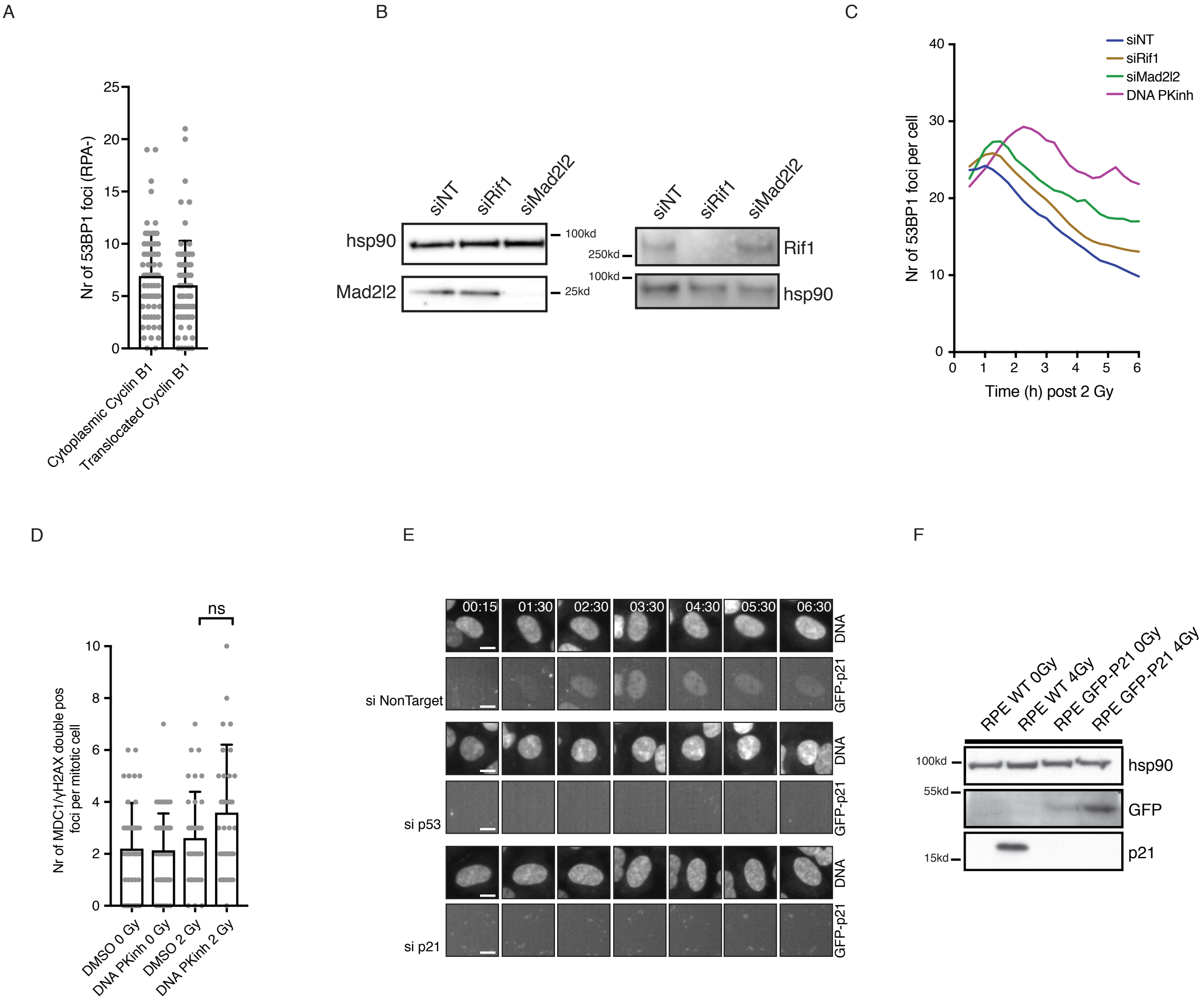

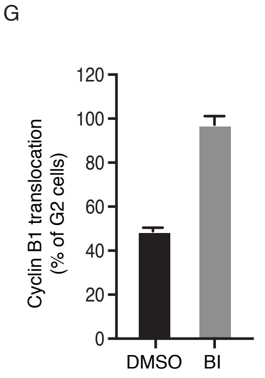

Supplementary figure 4. a) Number of 53BP1 foci that are negative for RPA staining, quantified 4 h after IR (2 Gy) in cells analysed in (Fig. 4c). **b)** Knock down control on WB for indicated siRNA transfections. **c)** Number of 53BP1 foci determined from live-cell imaging of RPE *CCNB1*^{YFP} – 53BP1^{mCherry} cells following IR (2 Gy). Mean lines from pooled individual cells, n>7 cells per condition. **d)** Number of MDC1 and γH2AX double positive foci in mitotic cells collected by nocodazole and fixed 9 h after IR in G2 phase. Cells in S phase at time of IR are excluded by EdU staining. n>35 cells pooled from two independent experiments + sd. **e)** Stills of RPE-1 cells with endogenously tagged ^{GFP}p21 show p21 induction upon IR (2 Gy). DNA is stained using sir-DNA. Scale bar 10 μm. **f)** Western blot showing homozygous expression of ^{GFP}p21 in RPE-1 cells. **g)** Percentage of G2 cells that translocate Cyclin B1 to the nucleus and exit the cell cycle following IR (2 Gy) in RPE *CCNB1*^{YFP} cells treated with DMSO or Plk1 inhibitor (BI). Average of two independent experiments + sd.

Supplementary Figure 5

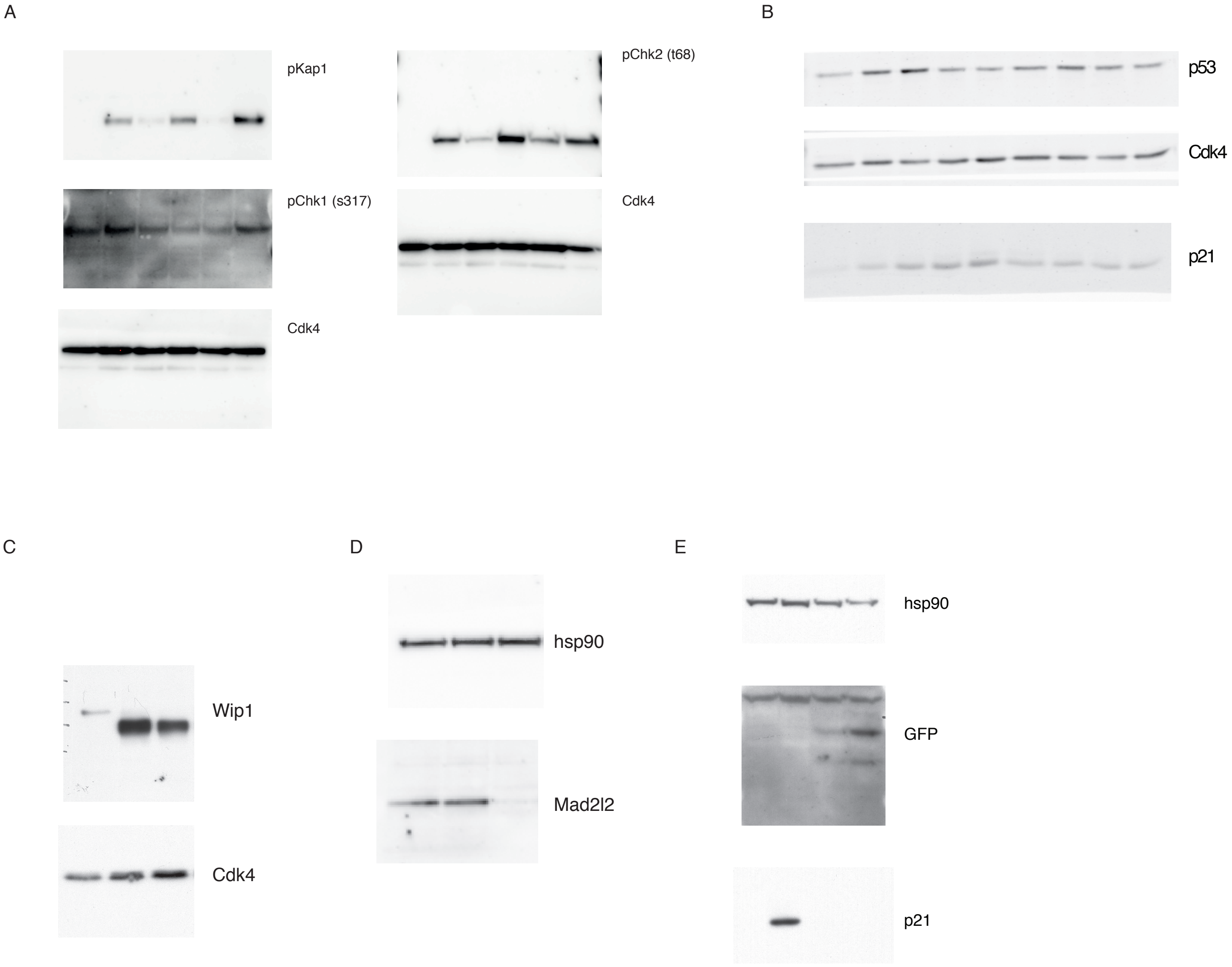

Supplementary figure 4. Uncropped western blots. a) Supplementary figure 2a. **b)** Supplementary figure 3a. **c)** Supplementary figure 3c. **d)** Supplementary figure 4b. **e)** Supplementary figure 4f.

Supplementary methods

Cell culture

To make BJhTert-Fucci cells, HEK293 cells were transfected with Fucci constructs[1] using X-tremeGENE (Roche) according to manufacturer's protocol. BJhTert cells were were infected after 2 days for 24 h and double positive Fucci cells were sorted by FACS two weeks later. BJhTert-Fucci were maintained in Dulbecco's Modified Eagle Medium/Nutrient Mixture F-12 (DMEM/F12, Gibco) supplemented with ultraglutamine, penicillin/streptomycin and 10% fetal calf serum.

Western Blot

For western blot analysis, equal amounts of proteins were separated by SDS–polyacrylamide gel electrophoresis followed by semi-dry transfer to a nitrocellulose membrane. Membranes were blocked in 5% milk in PBS-T for 1 hour at room-temperature before overnight incubation with primary antibody in PBS-T with 3% BSA at 4°C. Membranes were washed three times with PBS-T followed by incubation with secondary antibody in PBS-T with 5% milk for 2 hours at room-temperature. Antibodies were visualized using ECL (GE Healthcare). Uncropped western blot scans can be found in Suppl. Fig. 5.

1. Sakaue-Sawano, A. *et al.* Visualizing spatiotemporal dynamics of multicellular cell-cycle progression. *Cell* **132,** 487–498 (2008).
